# Changes in healthcare costs and survival in the era of immunotherapy and targeted systemic therapy for melanoma.

**DOI:** 10.23889/ijpds.v7i3.1828

**Published:** 2022-08-25

**Authors:** Paul Nguyen, Sarah Bateni, Antoine Eskander, Soo Jin Seung, Nicole Mittmann, Kelvin Chan, Nicole Look-Hong, Timothy Hanna

**Affiliations:** 1 ICES Queen's; 2 Division of General Surgery, University of Toronto; 3 Department of Otolaryngology, Sunnybrook Health Sciences Centre; 4 HOPE Research Centre; 5 CADTH; 6 Sunnybrook Research Institute; 7 Division of Cancer Care and Epidemiology, Cancer Research Institute at Queen's University

## Objectives

Melanoma treatment has evolved over the past decade with the adoption of adjuvant and palliative immunotherapies and targeted therapies and changes in use of sentinel node biopsy. The impact on real-world healthcare costs and outcomes is uncertain. Here we examine changes in healthcare costs and survival using administrative data.

## Approach

Using data from the universal healthcare system in Ontario, Canada, we examine a propensity-matched, retrospective cohort of patients aged 20+ years with Stage I-IV invasive cutaneous melanoma from 2018-2019 compared with those from 2007-2012. The primary outcomes were public payer’s mean healthcare per-person costs, and overall survival (OS). Costs were estimated with an established case-mix and claim-based costing algorithm for Ontario, in which person-level costs are allocated for the various healthcare utilizations over time. Standardized mean differences were used to compare costs, and the log-rank test and Cox regression were used to compare survival among stage-stratified, propensity-score matched cohorts.

## Results

We identified 1,138 patients with melanoma from 2018-2019 and 7,654 from 2007-2012. After stage stratification and propensity-matching (N=1,101 per cohort), sentinel lymph node biopsy (62.3% vs. 43.4%) and systemic therapy use (27.3% vs. 12.5%) were more frequent in 2018-2019 compared to 2007-2012. 2018-2019 patients had greater mean healthcare (including systemic therapy) costs compared to 2007-2012 with Stage II ($27,835 vs. $21,179), III ($90,508 vs. $46,242) and IV disease ($118,398 vs. $46,500). There was a seven-to-twelve-fold increase in mean systemic therapy costs for treated patients with Stage III ($68,207 vs. $9,832) and IV disease ($80,905 vs. $6,883). OS was greater in 2018-2019 versus 2007-2012 (2-year OS: 87.8% [95% Confidence Interval {CI}: 85.8-89.6%] vs. 83.7% [95% CI: 81.3-85.7%]; Hazard Ratio {HR}: 0.72 [95% CI: 0.59-0.89]; p<0.05).

## Conclusion

These real-world data highlight trade-offs with adoption of new effective systemic therapies for melanoma, with a greater economic burden to the healthcare system but an associated improvement in survival. Such evolving paradigm changes may prompt dynamic evaluations of healthcare resources and policies to ensure cancer care is sustainable.

